# The Odontological Society of Great Britain

**Published:** 1883-05

**Authors:** 


ARTICLE V.

### THE ODONTOLOGICAL SOCIETY OF GREAT BRITIAN.

The Fifth Ordinary Monthly Meeting of this Society for the present session was held at 40, Leicester Square, on Monday evening, March 5th, the chair being taken by the President, Dr. JOSEPH WALKER.

Mr. C. J. Wallis showed several models of peculiar cases among them being one with a peculiar abrasion in the upper jaw, one with three central incisors fully developed ; another with a supernumerary central in an upper jaw, also supernumerary central in lower jaw ; a model of the mouth of a lady aged 57 years who had recently erupted a canine ; also a model of a case of fibrous tumor from a patient who objected to any operation being performed, but the growth entirely disappeared by a special method of treatment by electrolysis.

Mr. Ackery showed a boy who in 1877, when five years old, had two supernumerary temporary teeth situate between and behind the lateral and canine, one on each side of the lower jaw. At present between the two canines in the

---

*Those who recognize the presence of germs in decayed teeth as well proved seem to be split up into the germ-inflammation adherents (Dr. Abbott ?) ; acid-germ, Dr. Miller ; germatony, Drs. A. & B. ; while others Dr. Stockwell, etc., consider the germs as most important, and the other changes as more secondary.

lower jaw there were eight teeth, two being temporary. He had no doubt but that there were successors to those two temporary teeth; Similar cases have been reported, one by Mr. Coleman in 1875, and Mr. C. Rogers in 1877.

Mr. J. S. Turner showed models of the jaws and the lower jaw of the girl whose remains were left in a box at Messrs. Carter, Paterson's. There was nothing of professional interest in the case, but as a great stir had been made respecting the matter he thought it would be well for them to know a little of the case.

Mr. Hutchinson showed, for Mr. Cornelius, a model of a case in which there was a perfectly formed supernumerary temporary incisor, the patient being three years and three months old.

The adjourned discussion upon the Etiology of Dental Caries having been postponed.

The President called upon Mr. Thorogood for his Paper on "Therapeutic Agents for the promotion of Osseous Development."

Having referred to the study and the relation of growth and nutrition, he said it was from lack of proper nourishment that certain organs of the body were apt to fail in function and waste in structure. The maintenance of the healthy nutrition of the human race was a work which concerned every department of medical, surgical and physiological science. The Public Health officer, the Physician and the Surgeon, were all called upon to labor to this end in harmonious concord, and benefits might be looked for as the result of a good understanding and co-operation between the Physician and the Dental Surgeon.

As an illustration of the important relation of the Dental art to that of Physic, he related the following case :—a gentleman, about twenty years ago, became such a martyr to dyspepsia that powders of bismuth and soda were regular concomitants of all his meals, and one of the highest medical authorities of the day pronounced the patient to be affected with cancer of the stomach, and prophesied that this maladay would soon end his days. A well known dental surgeon, however, begged the patient to allow him to remove some old roots of teeth and to fit him with a good set of artificial teeth. This proposal seemed almost a mockery to a man who had just been assured that he was gradually sinking from an inevitably fatal malady, but it was acted upon, with the result that the patient soon regained his digestive power, and was alive at the present day, a fairly vigorous man of 80 years of age.

The cause of wasting and failing nutrition was, however, not always so obvious or so easily remediable as in that case. In young and growing subjects especially, failure or perversion of nutrition might occur of so serious a nature as to give cause for great anxiety. For the promotion of osseous development, the use of mineral food for the nutrition of these tissues was a necessity. On the other hand the mere administration of the necessary lime salts was by no means the only thing to be considered in striving to improve osseous development. This was well seen in the disease called rickets, in which the evident deficiency of lime salts in the bones was accompanied by an elimination of from four to six times the normal amount of lime salts in the urine ; showing that the lime salts instead of being deposited in the bones were being excreted in the urine. This was said to be due to the presence of an excess of lactic acid in the system, and many of the symptoms of rickets favored this explanation. The child generally suffered from acid dyspepsia, sour-smelling perspiration, and sour-smelling offensive motions, etc. The recognition of a condition of acid dyspepsia, with gastro-intestinal irritation, in young children was very important, and every dental surgeon should have his eyes open to observe and detect it ; for it was a condition which undermined the results of his best work, and brought annoyance and vexation to parent and child, as well as to himself. The child's breath had a sour smell, the tongue was covered with white or yellowish fur, red papillæ showing through it, appetite often voracious, and the bowels confined or irregular. To give a big-bellied, pale-faced child in this condition phosphate of lime and iron would only make him more uncomfortable. But give him alkaline aperients, regulate his diet, cut of excess of starch and sugar, order exercise, salt water baths, etc., and then administer the specific remedies indicated. The child will thus be brought into a condition in which the Dental Surgeon will be able to work on the decayed molars with some prospect of his work remaining a lasting proof of his skill and dexterity.

Diet was a very important matter in these cases. The child must be taught to eat slowly. Brown or whole-meal bread and Scotch oatment generally agree well with children, and "seconds" flour was preferable to the best white on account of the larger proportion of phosphates it contains. Phosphate of lime might be given in doses of two or three grains twice daily as powder, or dissolved by the aid of a few drops of hydrocloride or phosphoric acid; but the remedies he had found most useful were the soluble hypophosphite of lime and the chloride of calcium. Either of these might be given in doses of two or three grains in glycerine and water with very beneficial effect. Lactophosphate of lime was another soluble lime salt which had proved valuable; whilst the ordinary lime-water and the saccharated solution of lime were specially useful in cases of irritable stomach, checking acidity and vomiting. Many children were now brought up on cow's milk, and it was important to observe the differences between this and human milk. To make cows' milk digestible, two parts of lime-water, or barley water, should be added to three of milk, and to each six ounces of the mixture a teaspoonful of sugar of milk should be added. The dilution with barley water prevented the casein coagulating in large indigestible masses. Asses' milk more nearly stimulated human milk. By observing such principles as he had indicated the good development of the bones and teeth would be promoted.

Mr. Coleman said he would just mention a case which corroborated a statement by Dr. Thorogood. A patient attended St. Bartholomew's Hospital for supposed cancer in the stomach, but it was afterwards believed to be chronic dyspepsia arising from imperfect mastication of food. The man had his teeth attended to, and quite recovered from his illness which, in the opinion of certain practitioners, was of the serious nature mentioned. They required to give something that would make the patient's teeth stronger and the material of the teeth harder; but there were so many things which would not be assimilated.

Mr. Sewill thought that Dr. Thorogood's paper opened up a wide subject. When enamel was once formed there was very little use in employing therapeutical agents to improve its structure—the treatment must come prior to that time. With regard to the question of dental deterioration, they now knew that hereditary syphilis and other diseases produced a marked mal-formation of the teeth. There was an apparently anomalous fact that people in the north (Yorkshire) with huge frames had poor teeth; it seemed to him that there was some special cause in deteriorating the structures of the teeth, while the bones were not so affected.

Mr. Stocken thought there was one point they should bear in mind, that the largest proportion of teeth they took out were first permanent molars, and that calcification of that tooth began *in utero*. It seemed to him to be a question more for the Medical man rather than the Dental Surgeon, as this aspect of preventive treatment must be with a mother during pregnancy. Therefore the medical practitioner should give advice to the mother as to diet, dress, etc., so that the dental organs should have proper development.

Mr. Cunningham, with reference to the remarks of the last speaker, thought a good deal might be done by proper advice in the matter. The subject had been well discussed by American Dental Societies.

Mr. Hele spoke of his experience with cloride of calcium, having watched its good effects in one of his own children.

Dr. Thorogood having replied, Mr. N. Stevenson and Mr. C. J. Wallis demonstrated the use of their electric lights, after which the meeting was adjourned.—*London Dental Review.*

